# Retraction: Variability in Bracing Design and Bracing Protocols for the Non-Operative Management of Legg-Calvé-Perthes Disease: A Scoping Systematic Review

**DOI:** 10.7759/cureus.r262

**Published:** 2026-11-04

**Authors:** Shubham Srivastava, Ajeet K Yadav, Rohit Shukla, Suresh Chand, Ajit Singh, Kanupriya Tiwari

**Affiliations:** 1 Orthopedics, Institute of Medical Sciences, Banaras Hindu University, Varanasi, IND; 2 Orthopedics, Autonomous State Medical College, Amethi, Amethi, IND; 3 Orthopedics, RKDF Medical College Hospital &amp; Research Centre, Bhopal, Bhopal, IND; 4 Pediatric Orthopedics, King George's Medical University, Lucknow, IND; 5 Microbiology, Institute of Medical Sciences, Banaras Hindu University, Varanasi, IND

This article has been retracted by the Editor-in-Chief due to concerns regarding the authorship and origin of the manuscript. After publication, information was received that called the published authorship and author contributions into question, and the authors were unable to provide records supporting the review. The authors do not agree with the decision to retract.

